# Rehabilitation after critical care: using audit to guide changes in practice, a multidisciplinary (MDT) approach

**DOI:** 10.1186/cc9951

**Published:** 2011-03-11

**Authors:** N Glasby, E Blake, D Green

**Affiliations:** 1 Kings College Hospital, London, UK

## Introduction

To audit the holistic assessment and treatment planning of critical care patients of more than 5 days stay, in line with the National Institute of Clinical Excellence (NICE) guidelines published in March 2009. The guidelines state that each patient should have a full comprehensive assessment and reassessment of all physical and nonphysical potential problems, individual goal-setting and documented communication between patient, MDT and family members.

## Methods

An audit form was developed from NICE guidelines and piloted with 10 patients, feedback on the audit form from the staff was then collected and the audit form amended as necessary. A sample of patients was identified (10% of 2008/2009 admissions of >5 days) and the first 10 sets of notes were assessed for inter-rater reliability between the staff collecting the information (doctors, occupational therapists, physiotherapists, nurses and speech and language therapists). The results were then compiled and new documentation developed to prompt consideration of potential physical and nonphysical problems. Weekly MDT rehabilitation ward rounds and goal-setting meetings were also commenced. A repeat audit using the same tool is to commence in December 2010/January 2011 with a second sample planned for June 2011/July 2011 in line with original audit samples.

## Results

Physical problems were comprehensively assessed in 100% of the sample population; however, there was little evidence of assessment of potential nonphysical problems in most patients. There was poor documentation of information-giving to patient relatives in all aspects of their care, particularly around goal-setting and social aspects of care. Transition from critical care to the ward was highlighted as an area to be improved, with poor information provision to the ward and to the patient/carer.

## Conclusions

Following the initial audit, in order to resolve the highlighted issues several initiatives were put in place: a rehabilitation ward round was commenced with weekly MDT goal-setting, a psychosocial history form was introduced along with a critical care MDT assessment tool. We are now beginning to re-audit following these changes in practice. Subjectively, collaborative working has enhanced patient care by optimising communication across the whole MDT. At the time of the conference 50% of the second audit will be completed, which will give some indication of the impact of our change in practice.
